# High Uric Acid Induces Insulin Resistance in Cardiomyocytes *In Vitro* and *In Vivo*

**DOI:** 10.1371/journal.pone.0147737

**Published:** 2016-02-02

**Authors:** Li Zhi, Zhu Yuzhang, Huang Tianliang, Ichiro Hisatome, Tetsuya Yamamoto, Cheng Jidong

**Affiliations:** 1 Department of Internal Medicine, The First Affiliated Hospital of Shantou University Medical College, Shantou, Guangdong, China; 2 Department of Internal Medicine, The Second Affiliated Hospital of Shantou University Medical College, Shantou, Guangdong, China; 3 Department of Internal Medicine, Chaozhou People’s Hospital, Chaozhou, Guangdong, China; 4 Department of Internal Medicine, The Second Hospital of Jiaxing City, Jiaxing, Zhejiang, China; 5 Division of Regenerative Medicine and Therapeutics, Institute of Regenerative Medicine and Biofunction, Graduate School of Medical Sciences, Tottori University, Yonago, Japan; 6 Department of Internal Medicine, Hyogo College of Medicine, Nishinomiya, Hyogo, Japan; Université catholique de Louvain, BELGIUM

## Abstract

Clinical studies have shown hyperuricemia strongly associated with insulin resistance as well as cardiovascular disease. Direct evidence of how high uric acid (HUA) affects insulin resistance in cardiomyocytes, but the pathological mechanism of HUA associated with cardiovascular disease remains to be clarified. We aimed to examine the effect of HUA on insulin sensitivity in cardiomyocytes and on insulin resistance in hyperuricemic mouse model. We exposed primary cardiomyocytes and a rat cardiomyocyte cell line, H9c2 cardiomyocytes, to HUA, then quantified glucose uptake with a fluorescent glucose analog, 2-NBDG, after insulin challenge and detected reactive oxygen species (ROS) production. Western blot analysis was used to examine the levels of insulin receptor (IR), phosphorylated insulin receptor substrate 1 (IRS1, Ser307) and phospho-Akt (Ser473). We monitored the impact of HUA on insulin resistance, insulin signaling and IR, phospho-IRS1 (Ser307) and phospho-Akt levels in myocardial tissue of an acute hyperuricemia mouse model established by potassium oxonate treatment. HUA inhibited insulin-induced glucose uptake in H9c2 and primary cardiomyocytes. It increased ROS production; pretreatment with N-acetyl-L-cysteine (NAC), a ROS scavenger, reversed HUA-inhibited glucose uptake induced by insulin. HUA exposure directly increased the phospho-IRS1 (Ser307) response to insulin and inhibited that of phospho-Akt in H9C2 cardiomyocytes, which was blocked by NAC. Furthermore, the acute hyperuricemic mice model showed impaired glucose tolerance and insulin tolerance accompanied by increased phospho-IRS1 (Ser307) and inhibited phospho-Akt response to insulin in myocardial tissues. HUA inhibited insulin signaling and induced insulin resistance in cardiomyocytes *in vitro* and *in vivo*, which is a novel potential mechanism of hyperuricemic-related cardiovascular disease.

## Introduction

Uric acid (UA) is the final product of purine metabolism. Elevated serum UA levels have been identified as a potential risk factor for gout, abnormal glucose metabolism, dyslipidemia and hypertension in the clinic but also strongly associated with cardiovascular diseases, including ischemic heart disease and heart failure [1–5]. Recent clinical studies have revealed that increased serum UA level is an independent risk factor for insulin resistance, and basic studies have showed that high uric acid (HUA) induced insulin resistance in liver, muscle and adipocytes. Measurements of insulin-stimulated glucose disposal are prognostic markers of heart failure [6–7]. However, direct evidence of how HUA affects insulin resistance in cardiomyocytes and a pathological mechanism to explain the association of HUA and cardiovascular disease remains to be clarified.

HUA levels increase oxidative stress in multiple cells [8–11]. Our previous study showed increased reactive oxygen species (ROS) production with HUA treatment in hepatocytes and pancreatic β cells [8–9]. Increased ROS levels are involved in insulin resistance. Although a pivotal role of HUA in insulin resistance in cardiac dysfunction is not yet established, increasing evidence has suggested that oxidative stress plays a causal role in the cardiac complications of insulin resistance, and overgenerated ROS and insulin resistance may both be implicated in cardiac dysfunction [12].

At the molecular level, insulin resistance is characterized by an impaired insulin-activated insulin receptor/insulin receptor substrate/phosphoinositide 3-kinase/protein kinase B (IR/IRS-PI3K-Akt) pathway, the major player in the metabolic action of insulin, which leads to suppressed insulin-induced glucose uptake in insulin-sensitive organs, including the heart [6,13]. IRS1 (Ser307) is centrally located within the insulin signaling pathway. Phosphorylation of IRS1 at Ser 307 has been extensively examined, in particular as part of a feedback loop in insulin signaling but also as input from other signaling pathways. Phosphorylation of IRS1 at Ser307 is of interest because it has been implicated as a causative mechanism of insulin resistance, as part of negative or positive control signals. The Akt family of intracellular Ser/Thr kinases regulates both cardiac growth and metabolism [14–15]. Akt regulates insulin-stimulated glucose uptake downstream of PI3K in cardiomyocytes [15]. However, whether HUA interferes with IRS-PI3K-Akt signaling in cardiomyocytes remains unclear.

In the present study, we investigated the effect of HUA on oxidative stress, insulin signaling and insulin resistance, as manifested by changes in ROS production, glucose uptake and phospho-IRS1 (Ser307) and -Akt activity in H9C2, primary cardiomyocytes and cardiac tissue from an acute hyperuricemia mice model. We examined whether the antioxidant N-acetyl-L-cysteine (NAC) can prevent HUA-induced insulin resistance, to examine the potential role of oxidative stress in the process.

## Materials and Methods

### Reagents

2-[N-(7-Nitrobenz-2-oxa-1,3-diazol-4-yl)amino]-2-deoxy-d-glucose (2-NBDG) was from Invitrogen (Carlsbad, CA, USA). 2,7,-dichlorodihydrofluorescein diacetate (DCFH-DA), UA and SP600125 were from Sigma (St. Louis, MO, USA). Anti-phospho-Akt (Ser473) and anti-Akt antibodies were from Bioworld (St. Louis Park, MN, USA). Anti-phospho-IRS1 (Ser307) and anti-IRS1 (Ser307) antibodies were from Millipore (Billerica, MA). Anti-phospho-IR (Tyr1361), antibodies against insulin receptor (IR), Anti-phospho-insulin-like growth factors-1 (IGF1R, Tyr1161), c-Jun N-terminal kinase (JNK), JNK and rabbit GAPDH were from Abcam. NAC was from ENZO Life Sciences (Farmingdale, NY, USA). All chemical reagents were of analytical grade. For primary buffer, UA stock solution was prepared at 15 mg/ml in 0.5 M NaOH. NAC stock solution was prepared at 500 mM in ultrapure water and final concentration was 5 mM.

### Cell culture and treatment

Cellular studies were conducted with H9c2 rat heart-derived embryonic myocytes (CRL-1446; American Type Culture Collection, Manassas, VA) incubated with DMEM or low-glucose DMEM supplemented with 10% fetal bovine serum (FBS), 100 U/ml penicillin G, 100 mg/ml streptomycin, and 2 mM L-glutamine. Primary cardiomyocytes were prepared from neonatal C57BL/6 mice and obtained as previously reported [16]. Cells were incubated at 37°C with 5% CO_2_ and 95% air. For all experiments, cells were plated in six-well plates at 2.0×10^5^ cells/ml. For HUA treatment, cells were incubated with 15 mg/dl HUA in fresh cell-culture medium for 24 h. Cells were incubated for the indicated times, then harvested for biochemical or molecular assays. All experiments were repeated at least 3 times.

### Fluorescence microscopy analysis of glucose (2-NBDG) uptake in cardiomyocytes

Glucose uptake of H9c2 cardiomyocytes was assessed by the fluorescent glucose analog, 2-NBDG. Briefly, cells were treated with low-glucose DMEM supplemented with free FBS for 24 h, then the medium was replaced with Krebs-Ringer-Bicarbonate (KRB) buffer containing insulin (100 nM; final concentration) and 2-NBDG (100 μM; final concentration) for 30 min at 37°C with fluorometry excitation and emission 485 and 535 nm, respectively.

### Flow cytometry of glucose (2-NBDG) uptake in cardiomyocytes

Cells were treated with low-glucose DMEM supplemented with free FBS for 24 h, then the medium was replaced with KRB buffer containing insulin (100 nM; final concentration) and 2-NBDG (100 μM; final concentration) for 30 min at 37°C. Free 2-NBDG was washed from cultures after treatment and 2-NBDG was measured by flow cytometry (BD Biosciences, San Jose, CA, USA) at excitation and emission 488 nm and 525 nm, respectively. For each sample, 20,000 events were acquired in the regions of the FSC x SSC plot. Data analysis involved flow cytometry software (BD Biosciences, San Jose, CA, USA), and the normalized median fluorescence intensities were calculated as the ratios in median fluorescence intensities between treated and untreated cells. Statistical analysis involved use of GraphPad Instat (GraphPad Software Inc., La Jolla, CA, USA) by ANOVA followed by Tukey-Kramer multiple comparisons test.

### Analysis of intracellular ROS levels

Cells were subcultured in 6-well plates (2.0×10^5^ cells/well), allowed to attach for 24 h, exposed to HUA for 24 h, and stained with 10 mM DCFH-DA for 30 min at 37°C as described [17]. Stained cells were imaged by fluorescence microscopy and analyzed by flow cytometry at excitation and emission 530 and 480 nm, respectively.

### Animals

This study was carried out in strict accordance with the recommendations in the Guide for the Care and Use of Laboratory Animals of the National Institutes of Health. The protocol was approved by the Committee on the Ethics of Animal Experiments of the University of Shantou (Permit Number: SYXK2007-0079). Eight-week-old male C57BL/6J mice (20±2 g) were from Vital River Laboratories (Beijing) and housed in the Laboratory Animal Center of Shantou University Medical College. All surgery was performed under sodium pentobarbital anesthesia, and all efforts were made to minimize suffering.

Mice were fed a standard diet and maintained in individual cages with routine light–dark cycles and allowed to adapt to the laboratory environment for 1 week. Animals were anesthetized by injecting sodium pentobarbital (50 mg/kg intraperitoneally). Single ventricular myocytes were isolated as described [18].

### Hyperuricemia mice model and treatment

Ten-week-old male C57BL/6J mice were randomly assigned to 2 groups (n = 4 each) for treatment: control and HUA (n = 4). For HUA treatment, following an 18-h overnight fast, mice received an intraperitoneal (i.p.) injection of potassium oxonate (300 mg/kg) and intragastric administration of hypoxanthine (500 mg/kg) to create the acute hyperuricemia model for 1–2 h. The volume of drug was based on body weight measured immediately before each dose. Then serum UA level was inspected at different times by the phosphotungstic acid method [19]. Glucose tolerance and insulin tests were performed as we previously described [8]. For control and HUA mice, insulin (2 U/kg) was injected 10 min before they were killed by CO_2_ inhalation, and left ventricle cardiac muscle tissue was excised. All tissue samples were immediately stored in liquid nitrogen.

### Western blot analysis

Cells were lysed, sonicated and homogenized in radioimmunoprecipitation assay (RIPA) buffer, supplemented with protease inhibitors (1 mmol/l phenylmethanesulfonyl fluoride, PMSF) and phosphatase inhibitors (phosphatase inhibitor mixture I). The supernatant protein concentration was determined by use of the BCA Protein Assay Kit (Pierce, IL, USA), then equal amounts of total protein underwent 10% SDS-PAGE and were transferred to polyvinylidene difluoride membranes (Millipore, Shanghai), which were blocked with 5% non-fat milk and incubated with primary antibodies for phosphorylated and total (1:1000 dilution), then horseradish peroxidase-conjugated secondary antibody (1:10,000 dilution). An enhanced chemiluminescence kit (Pierce, IL, USA) was used for signal detection. Images of blots were acquired by a digital image processing system (Universal HoodII76S/0608, Bio-Rad, Hercules, CA) and quantified by use of Quantity One (Bio-Rad).

### Statistical Analysis

Data are described with mean ± SD and were analyzed by use of SPSS (SPSS Inc., Chicago, IL) with unpaired Student’s *t* test or one-way ANOVA. Significant differences were determined by Duncan’s multiple range tests. Results were considered statistically significant at P < 0.05.

## Results

### HUA suppressed insulin-induced glucose uptake in H9c2 cardiomyocytes

**Fig 1:**
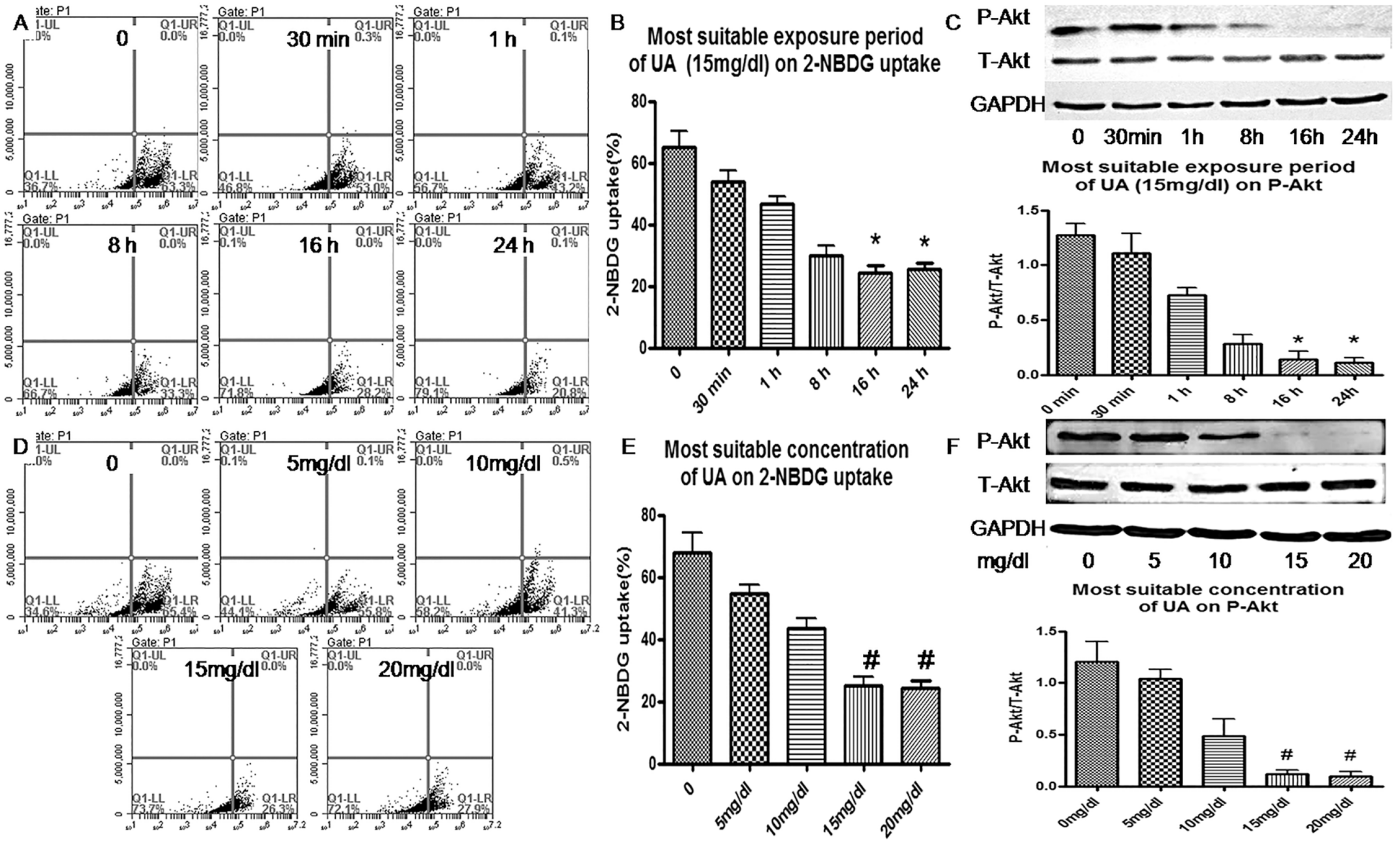
Effect of uric acid (UA) on 2-[N-(7-Nitrobenz-2-oxa-1,3-diazol-4-yl)amino]-2-deoxy-d-glucose (2-NBDG) uptake and Akt phosphorylation in H9c2 cardiomyocytes. (A, B, D, E) Flow cytometry of cells pretreated with UA (0, 5, 10, and 15 mg/dl) or 0, 30 min and 1, 8, 16 and 24 h, then assayed for insulin-stimulated 2-NBDG uptake. (C, F) Western blot analysis of phosphorylated and total Akt levels. *P<0.05 vs. 0, 30 min, and 1 h. #P<0.05 vs. 0, 5, and 10 mg/dl. GAPDH was a normalization control.

**Fig 2:**
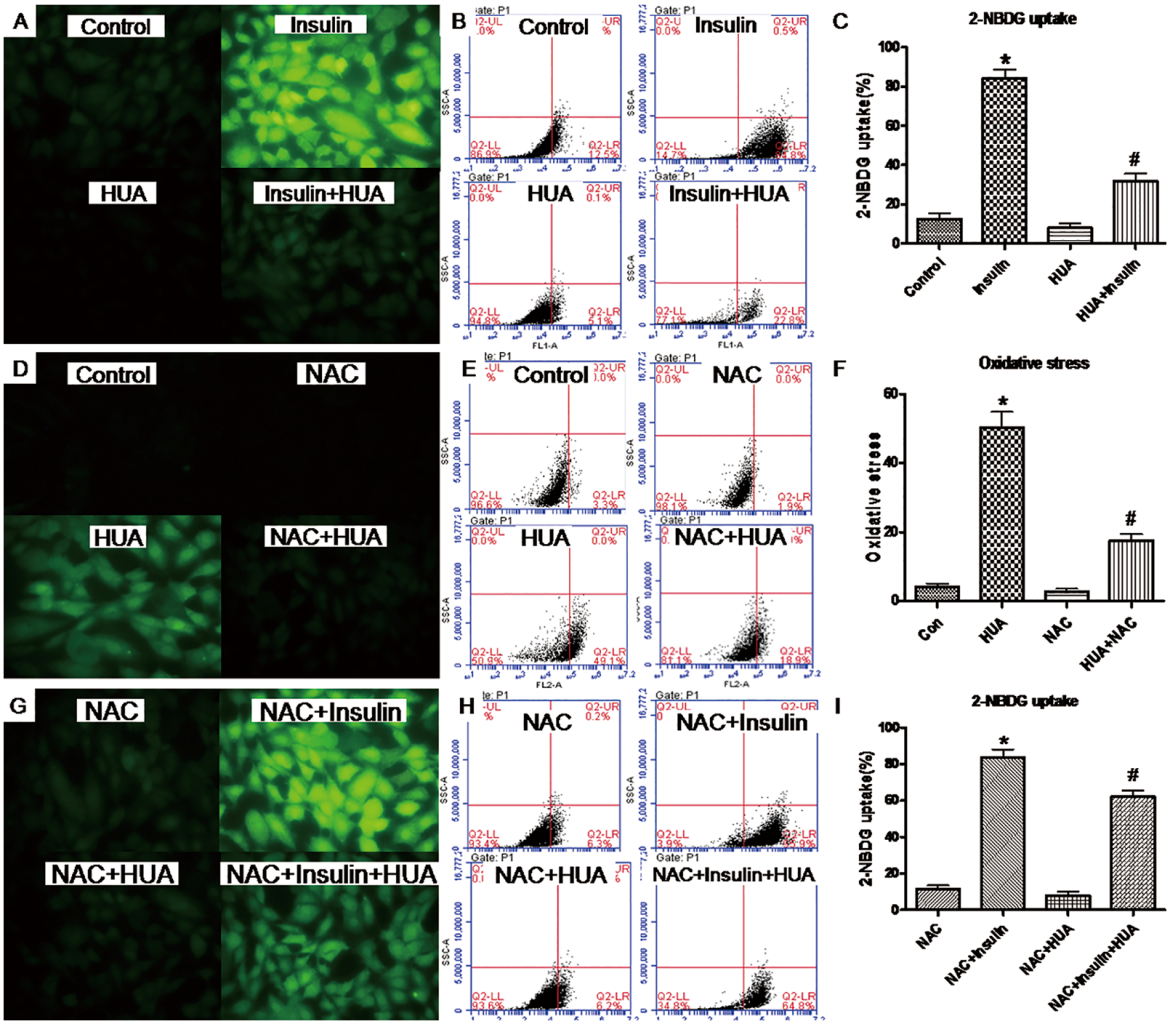
(A-C) Effect of HUA on 2-NBDG uptake in H9c2 cardiomyocytes. Cells were pretreated with HUA, then underwent basal or insulin-stimulated 2-NBDG uptake assay detected by fluorescence microscopy (A) and analyzed by flow cytometry (B). (C) *P<0.01 vs. control, #P<0.01 vs. insulin and HUA. (D-E) Effect of HUA on ROS generation in H9c2 cardiomyocytes. Cells were co-incubated with HUA and stained with DCFH-DA for fluorescence microscopy (D) and analyzed by flow cytometry (E). (F) *P<0.01 vs. control, #P<0.01 vs. HUA and N-acetyline cystein (NAC). (G-I) Effect of HUA on 2-NBDG uptake in H9c2 cardiomyocytes via oxidative stress. Cells were pretreated with HUA with or without NAC, then underwent basal or insulin-stimulated 2-NBDG uptake assay detected by fluorescence microscopy (G) and flow cytometry (H). (I) *P<0.01 vs. NAC, #P<0.01 vs. NAC+HUA. Data are mean±SD from 3 separate experiments.

To determine whether HUA induces insulin resistance *in vitro*, H9c2 cardiomyocytes were pretreated with concentrations of UA for various times, then underwent insulin-stimulated 2-NBDG uptake and Akt phosphorylation assay (Fig 1A–1F). Insulin significantly increased 2-NBDG uptake and Akt phosphorylation in H9c2 cardiomyocytes, and pre-treatment with HUA (15 mg/dl) for 24 h suppressed the insulin-induced 2-NBDG uptake and Akt phosphorylation (Figs 1A–1F and 2A–2C). Therefore, HUA inhibited insulin-induced glucose uptake and induced insulin resistance in H9c2 cardiomyocytes.

### HUA induced oxidative stress in H9c2 cardiomyocytes

ROS level was higher in H9c2 cardiomyocytes with than without HUA treatment (Fig 2D–2F). Pretreatment with the antioxidant NAC partially reversed HUA-generated ROS (Fig 2D–2F), which suggests that HUA directly caused oxidative stress in H9c2 cardiomyocytes.

### HUA induces insulin resistance via oxidative stress in H9c2 and primary cardiomyocytes

**Fig 3:**
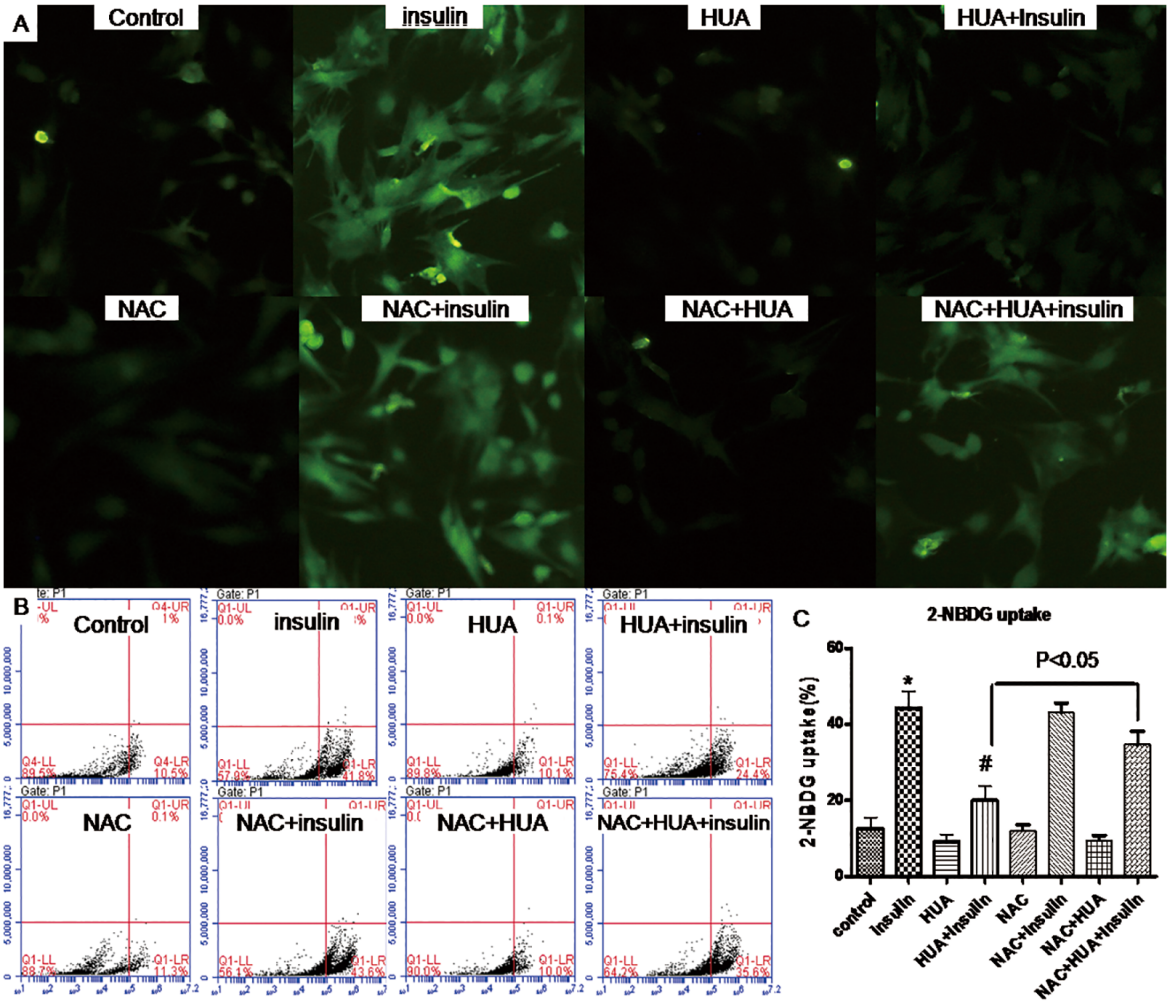
Effect of HUA on 2-NBDG uptake in primary cardiomyocytes via oxidative stress. Cells were pretreated with HUA with or without NAC, then underwent basal or insulin-stimulated 2-NBDG uptake assay detected by fluorescence microscopy (A) and analyzed by flow cytometry (B) (C). *P<0.01 vs. control, #P<0.01 vs. insulin.

Next, we tested whether treatment with HUA induced insulin resistance via oxidative stress in H9c2 and primary cardiomyocytes. Pretreatment with NAC reversed HUA-reduced 2-NBDG uptake induced by insulin (Figs 2G–2I and 3A–3C), so oxidative stress may play a key role in HUA-induced insulin resistance in H9c2 and primary cardiomyocytes.

### HUA increased phospho-IRS1 (Ser307) level but had no effect on IR expression in H9c2 cells

**Fig 4:**
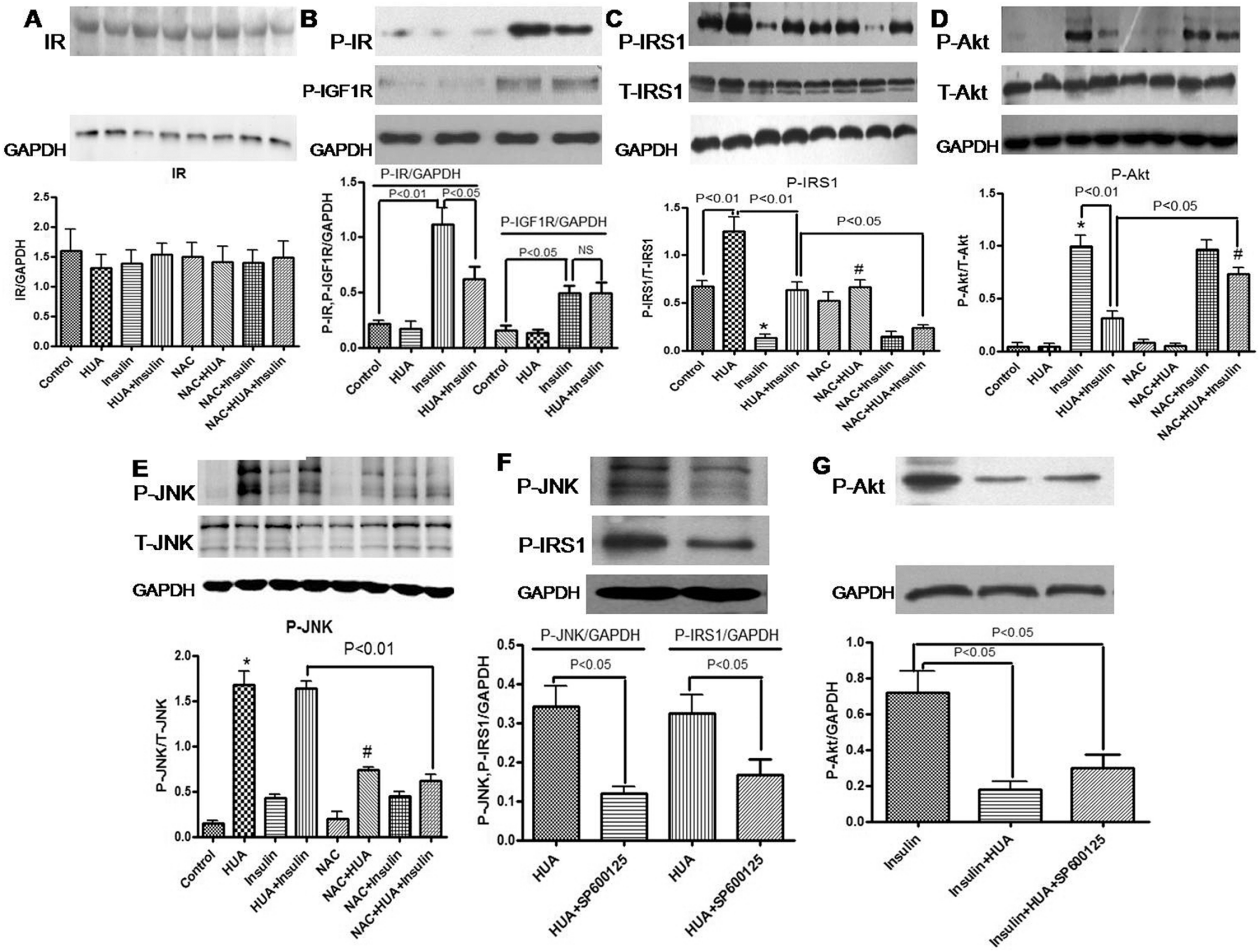
(A-E) Effect of HUA on IR (A), phospho-IR (Tyr1361) and phospho-IGF1R (Tyr1161) (B), total and phosphorylated levels of IRS1(Ser307) (C), Akt (D) and JNK (E) in H9c2 cardiomyocytes. Western blot analysis of cells pretreated with HUA with or without NAC, then examined for basal or insulin-inhibited expression. Data are mean±SD from 3 separate experiments. (C) *P<0.01 vs. control and HUA+insulin; #P<0.05 vs. HUA. (D) *P<0.01 vs. control and HUA; #P<0.01 vs. NAC+HUA. (E) *P<0.01 vs. control; #P<0.05 vs. HUA. (F-G) Effect of SP600125 on HUA-mediated phospho-JNK, phospho-IRS1(Ser307) and phospho-Akt in H9c2 cardiomyocytes.

As compared with other phosphorylated forms of IRS1 (Ser307), which activate insulin signaling, phospho-IRS1 (Ser307) inhibits insulin signaling. In this study, HUA exposure increased phospho-IRS1 (Ser307) level in H9c2 cardiomyocytes (Fig 4C) but had no effect on IR expression (Fig 4A). Furthermore, HUA reversed the insulin-inhibited level of phospho-IRS1 (Ser307) in H9c2 cardiomyocytes (Fig 4C).

To determine whether HUA-induced oxidative stress activated phospho-IRS1 (Ser307) signaling in H9c2 cardiomyocytes, we examined the effect of NAC treatment on HUA-induced phospho-IRS1 (Ser307) level. NAC neutralized the effect of HUA-induced phospho-IRS1 (Ser307) activity (Fig 4C), which demonstrates a critical role of oxidative stress in the effect of HUA on insulin resistance.

### HUA inhibiting phospho-IR (Tyr1361) on response to insulin led to insulin resistance but had no effect on phospho-IGF1R (Tyr1161) expression in H9c2 cells

Phospho-IR (Tyr1361) which mediates the pleiotropic actions of insulin. Binding of insulin leads to the activation of a main signaling pathway: the IRS-PI3K-Akt pathway, which is responsible for most of the metabolic actions of insulin. We examined whether HUA induces insulin resistance are insulin receptor-mediated or can be ascribed to activation of the IGF1R in H9c2 cells. In present study, insulin-induced phospho-IR (Tyr1361) level was significantly suppressed by HUA (Fig 4B). Next, we examined the role of IGF1R on HUA induces insulin resistance in H9c2 cells. Insulin-induced phospho-IGF1R level significantly but HUA did not affect insulin-induced phospho-IGF1R level (Fig 4B). Therefore, HUA induced insulin resistance in H9c2 cardiomyocytes was insulin receptor-mediated.

### HUA inhibiting phospho-Akt response to insulin led to insulin resistance via oxidative stress in H9c2 cardiomyocytes

Akt is a Ser/Thr protein kinase activated by insulin via a PI3K-dependent pathway in insulin signaling transduction. We examined whether HUA inhibited Akt phosphorylation with insulin stimulation in H9c2 cells. Insulin-induced phospho-Akt level was significantly suppressed by HUA (Fig 4D).

Next, we examined the role of the antioxidant NAC on HUA-inhibited Akt activity. NAC did not affect insulin-induced phospho-Akt level, but insulin-induced phospho-Akt level was inhibited by HUA treatment, which was reversed significantly by NAC treatment (Fig 4D). The expected increase in phospho-Akt level was inhibited by exposure to HUA, which was reversed by NAC treatment and demonstrates a profound effect of HUA on downstream insulin signaling via oxidative stress.

### HUA induced phospho-JNK expression, but JNK inhibition did not affect HUA-decreased glucose uptake stimulated by insulin in H9c2 cardiomyocytes

**Fig 5:**
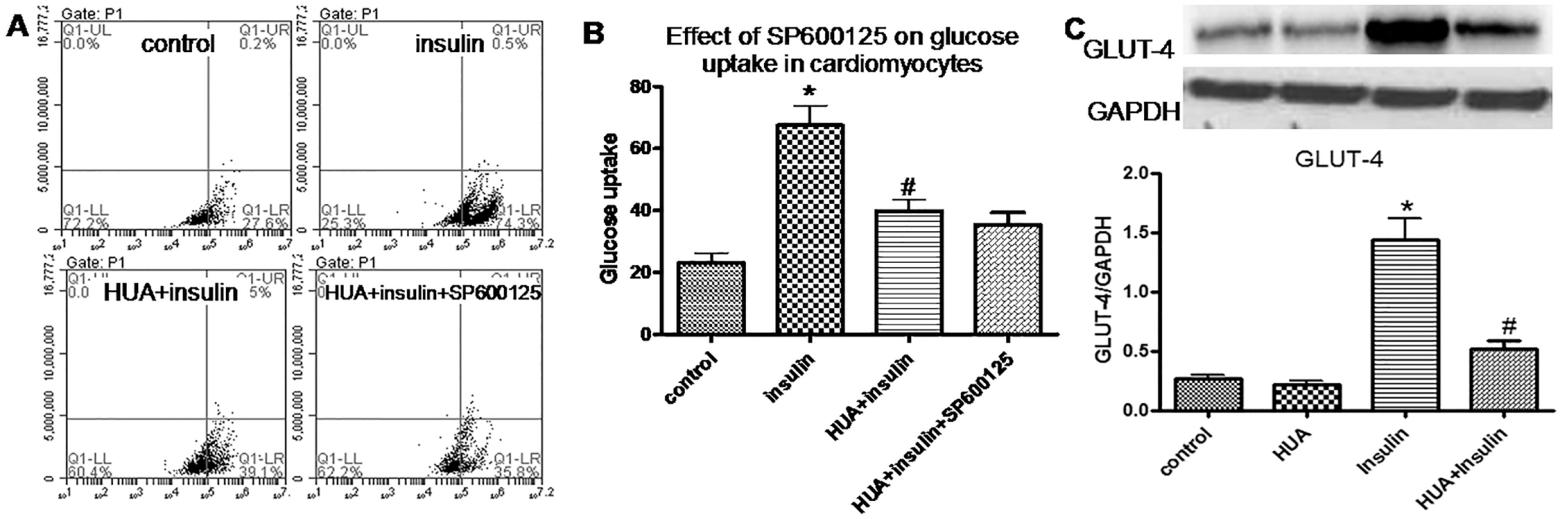
(A-B) Effect of SP600125 on HUA inhibiting 2-NBDG uptake stimulated by insulin in H9c2 cardiomyocytes. (B) *P<0.01 vs. control; #P<0.01 vs. insulin. (C) Effect of HUA on GLUT4 expression in H9c2 cardiomyocytes. (C) *P<0.01 vs. control and HUA; #P<0.01 vs. insulin.

HUA induced phospho-JNK expression, whereas insulin was a weak stimulator of phospho-JNK (Fig 4E). NAC neutralized the effect of HUA-induced phospho-JNK activity (Fig 4E), which indicates a key role of oxidative stress in the effect of HUA on phospho-JNK. Furthermore, after pretreatment with SP600125, a JNK inhibitor, the activation of phospho-IRS1 (Ser307) induced by HUA was reduced significantly (Fig 4F). However, SP600125 did not affect phospho-Akt (Fig 4G), nor did it change HUA-decreased glucose uptake stimulated by insulin (Fig 5A and 5B).

### High level of insulin increases GLUT4 translocation to the cell surface, which was significantly inhibited by HUA

To determine whether HUA inhibited glucose uptake in H9c2 cardiomyocytes by affecting GLUT4 expression, we examined the effect of insulin and HUA on GLUT4 expression in H9c2 cardiomyocytes. Treating H9c2 cells with insulin (100 nM for 30 min) significantly increased GLUT4 expression, which was significantly inhibited by HUA (Fig 5C). Therefore, HUA inhibited glucose uptake in H9c2 cardiomyocytes probably via GLUT4 expression.

### Hyperuricemia increased phospho-IRS1 (Ser307) level and inhibited phospho-Akt response to insulin in mouse cardiac tissue

**Fig 6:**
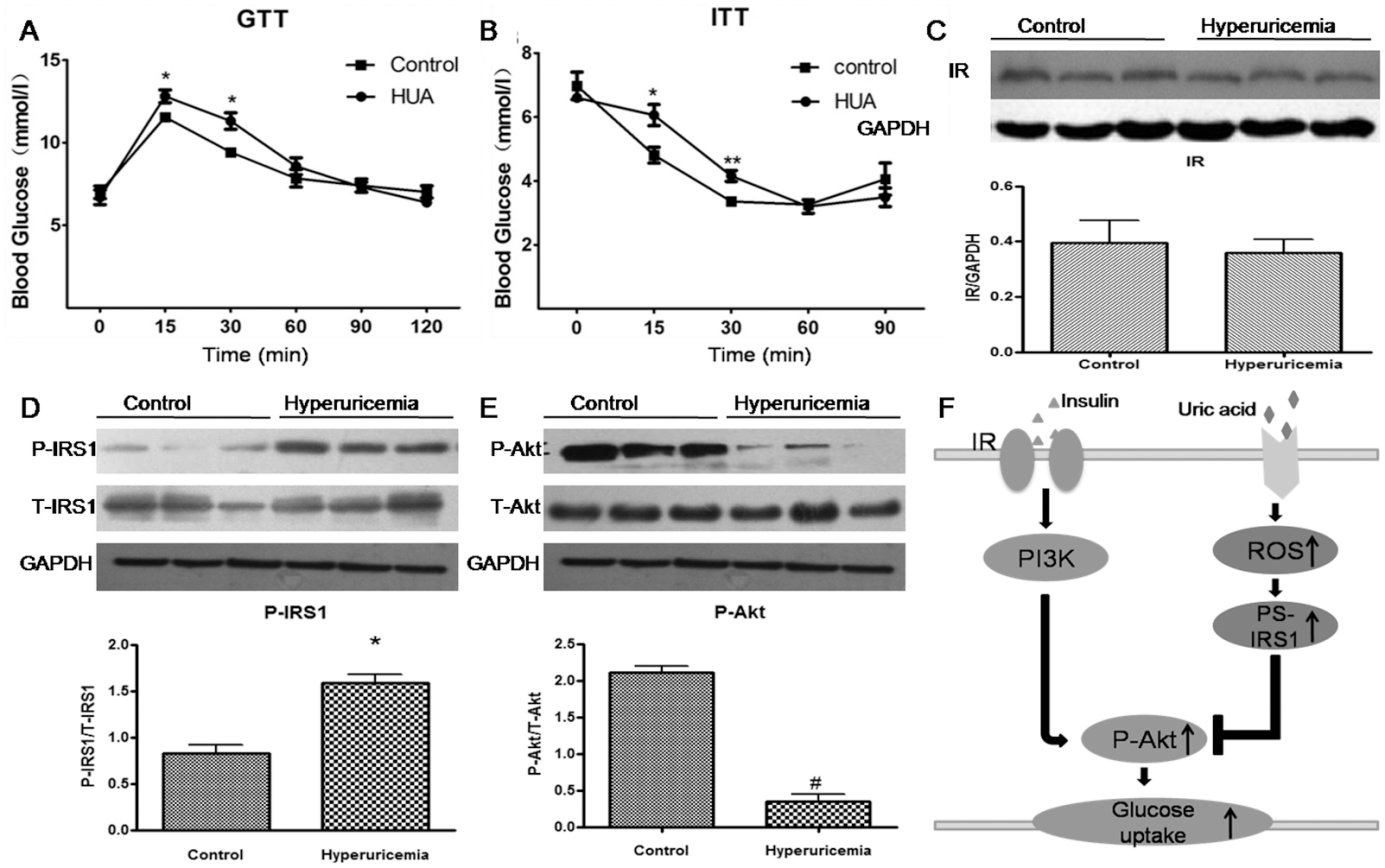
(A-B) Glucose tolerance test (GTT) (A) and insulin tolerance test (ITT) (B) in an acute hyperuricemic mice model (with HUA level). Data are mean±SD from 3 separate experiments. *P < 0.05, **p < 0.01 vs. control. (C-E) Western blot analysis of IR (C), phospho-IRS1 (Ser307) (D) and phospho-Akt (E) level in cardiac tissues. *P<0.01 vs. control. #P<0.01 vs. control. (F) Schematic representation of HUA-mediated insulin resistance in cardiomyocytes. Increased HUA-induced oxidative stress activates phospho-IRS1 (Ser307) level, which impairs Akt (Ser 437) phosphorylation, thus increasing acute insulin resistance in cardiomyocytes with HUA treatment.

As expected, serum UA levels in our mouse model were higher after than before hyperuricemia induction (113.81±17.98 vs 38.03±2.68 mg/l), which was consistent with patients with primary hyperuricaemia [20]. Our mouse model showed impaired glucose tolerance (Fig 6A) and insulin tolerance (Fig 6B) at 15 and 30 min after glucose or insulin injection with insulin resistance. We examined insulin signaling in cardiac tissues of the acute hyperuricemic mouse model. Insulin was intraperitoneally injected in hyperuricemic mice for 10 min, then cardiac tissues were obtained. Hyperuricemia increased the phospho-IRS1 (Ser307) level in mouse cardiac tissues (Fig 6D). Phospho-Akt level was lower in cardiac tissues of acute hyperuricemic mice than controls (Fig 6E), with no change in IR (Fig 6C), which demonstrates a profound effect of hyperuricemia on downstream insulin signaling *in vivo*.

## Discussion

In this study, we investigated the mechanism of HUA-induced insulin resistance in cardiomycytes. HUA may induce oxidative stress and play a causative role in the development of insulin resistance in cardiomyocytes (Fig 4). HUA-increased phospho-IRS1 (Ser307) level provides insight into one mechanism whereby HUA exposure can impair insulin signaling. In addition, phospho-Akt-mediated insulin signaling inhibited by HUA is linked to impeded glucose uptake and insulin resistance in cardiomyocytes. Antioxidants such as NAC could prevent the HUA-increased phospho-IRS1 (Ser307) level, -decreased phospho-Akt level and -inhibited glucose uptake in cardiomyocytes. Furthermore, a hyperuricemia mice model showed increased phospho-IRS1 (Ser307) and inhibited phospho-Akt levels with glucose intolerance and insulin resistance. HUA may indirectly inhibit insulin signaling and induce insulin resistance via oxidative stress in cardiomyocytes.

Serum HUA is strongly associated with many cardiovascular diseases such as heart failure [1–2,6], hypertension [4], and coronary artery disease [3,5], but a pathological mechanism to explain this association has been missing. HUA increases oxidative stress in pancreatic ß cells, adipocytes and mesangial cells [9–11]. Furthermore, oxidative stress is a major cause and acts as a major mediator of insulin resistance in many cell types, including cardiomyocytes [12, 21–24]. Insulin resistance is a risk factor for mortality in patients with heart failure, and cardiac oxidative stress often occurs with insulin resistance [6]. HUA worsens insulin resistance, which was confirmed in our recent study [8]. Here, we demonstrated that HUA could induce insulin resistance directly in cardiomyocytes via oxidative stress. Our results provide novel evidence for HUA as an independent risk factor for cardiovascular disease and a new explanation for how HUA inhibits glucose metabolism of cardiomyocytes and is associated with cardiovascular disease.

IR and IRS1 (Ser307) are crucial counterregulated mediators of the insulin signaling pathway. IR plays a critical role in the homeostasis of glucose metabolism by regulating GLUT4 translocation to the cell surface in adipose tissue and muscle. The IR and IGF-1R are very similar tetrameric glycoproteins omposed of two extracellular a and two transmembrane subunits, linked by disulphide bonds. In present study, insulin-induced phospho-IR (Tyr1361) level was significantly suppressed by HUA but HUA did not affect insulin-induced phospho-IGF1R (Tyr1161) level. Therefore, HUA induced insulin resistance in H9c2 cardiomyocytes was insulin receptor-mediated. Previous studies provided evidence to suggest that the localization of IRS1 to the intracellular membrane is important for efficient insulin signaling and biological responsiveness [25]. Phosphorylation of IRS1 (Ser307) is a representative molecular marker of insulin resistance. IRS1 Ser phosphorylation leads to decreased Tyr phosphorylation and increased proteasome-mediated degradation. Increased Ser phosphorylation of IRS-1 is a common finding in insulin resistance. In this study, the finding that HUA increased Ser phospho-IRS1 but had no effect on IR provides insight into a mechanism of HUA exposure impairing insulin signaling because phospho-IRS1 (Ser307) inhibited downstream insulin signaling: it reduced PI3K activity and the following phospho-Akt level.

Recent work suggests that phospho-Akt regulates cell metabolism, growth and survival, and cardiac growth and metabolism are coordinated by the integration of a complex array of extracellular and intracellular signals [26,14]. Moreover, activated phospho-Akt, a key mode of insulin-induced glucose uptake, regulates insulin-stimulated glucose uptake in adult mouse cardiomyocytes [27]. Our findings show that HUA can inhibit phosphor-Akt activation and glucose uptake induced by insulin in cardiomyocytes. HUA-induced phospho-IRS1 (Ser307) inhibits the activity of Akt to decrease glucose uptake and insulin resistance.

Previous studies have shown that JNK activation is associated with oxidative stress-induced insulin resistance by H2O2 in skeletal muscle [28]. In this study, directly exposing H9c2 cardiomyocytes to HUA increased JNK phosphorylation. Furthermore, we tested the effect of JNK inhibition in HUA-mediated phospho-IRS1 (Ser307). We found that in H9c2 cardiomyocytes, inhibition of JNK leads to a decrease in HUA-induced phospho-IRS1 (Ser307). However, JNK inhibition did not reverse the HUA-inhibited phospho-Akt and 2-NBDG uptake stimulated by insulin in H9c2 cardiomyocytes. This theoretical additive value assumes that JNK did not have a direct effect on oxidative-stress induced insulin resistance in cardiomyocytes, which was consistent with previous study in skeletal muscle and vascular smooth muscle cells [28–29]. Inhibition of JNK partially blocks HUA-mediated phospho-IRS1 (Ser307) in this study, which implicates that JNK may regulates other cellular processes mediated by IRS-1. Many reports have showed that IRS-1 proteins are involved in many other cellular functions, including protein metabolism, migration, apoptosis, and amino acid transport.

The change in expression of the insulin-stimulated glucose transporter GLUT4 could affect glucose uptake in the myocardial cells. However, the cardiomyocyte cell line H9C2 is relatively insensitive to insulin. A high level of insulin (100 nM for 30 min) increased glucose uptake by recruiting GLUT4 from intracellular vesicles to the plasma membrane of H9c2 cells [30]. To determine whether HUA inhibited glucose uptake by affecting GLUT4 expression in H9c2 cardiomyocytes, we further examined the effect of insulin and HUA on GLUT4 expression in H9c2 cardiomyocytes. Treating H9c2 cells with insulin (100 nM for 30 min) significantly increased GLUT4 expression, which was inhibited by HUA. Thus, HUA inhibited glucose uptake in H9c2 cardiomyocytes probably by affecting GLUT4 expression. Another study [31] showed that glucose and insulin levels could regulate glucose uptake in H9c2 myocardial cells by affecting GLUT4 expression and thus cell proliferation and cell function. Insulin levels could affect myocardial cell function by regulating GLUT4 expression. The effect of glucose and insulin on myocardial cell proliferation might be mediated by regulating GLUT4 expression. There may be a mechanism of hyperglycemia preaccommodation (HGPA) in myocardial cells mediated by regulation of GLUT4 expression.

Insulin is an important regulatory hormone that mediates energy uptake by increasing glucose uptake into cardiac tissues [27]. In this study, hyperuricemic mice showed significantly increased cardiac phospho-IRS1 (Ser307) and decreased phospho-Akt levels with glucose intolerance and impaired insulin resistance. HUA may change the energy metabolism by decreasing glucose uptake and increasing fatty acid utilization. As well, HUA-induced insulin resistance in cardiac tissue may contribute to cardiac dysfunction. We used an acute hyperuricemia mouse model, but human hyperuricemia is a chronic process. Further study with a chronic hyperuricemia mouse model may be needed to clarify the association of hyperuricemia and insulin resistance in heart tissue.

## Conclusions

In summary, HUA inhibited insulin signaling and induced insulin resistance in cardiomyocytes *in vivo* and cardiac tissue *in vitro*. We provide a novel potential mechanism for hyperuricemia associated with cardiovascular disease. However, further study is needed to confirm how HUA leads to abnormal energy metabolism in cardiomyocytes and its significance in heart disease.
